# Supplementary material for: Prompt Graft Cooling Enhances Cardioprotection during Heart Transplantation Procedures through the Regulation of Mitophagy
Source: Cells. 2021 Oct 27;10(11):2912. doi: 10.3390/cells10112912 (PMC8616468; doi:10.3390/cells10112912)
Supplement: Supplementary file 1 [file cells-10-02912-s001.zip › cells-1342870-supplementary.pdf]

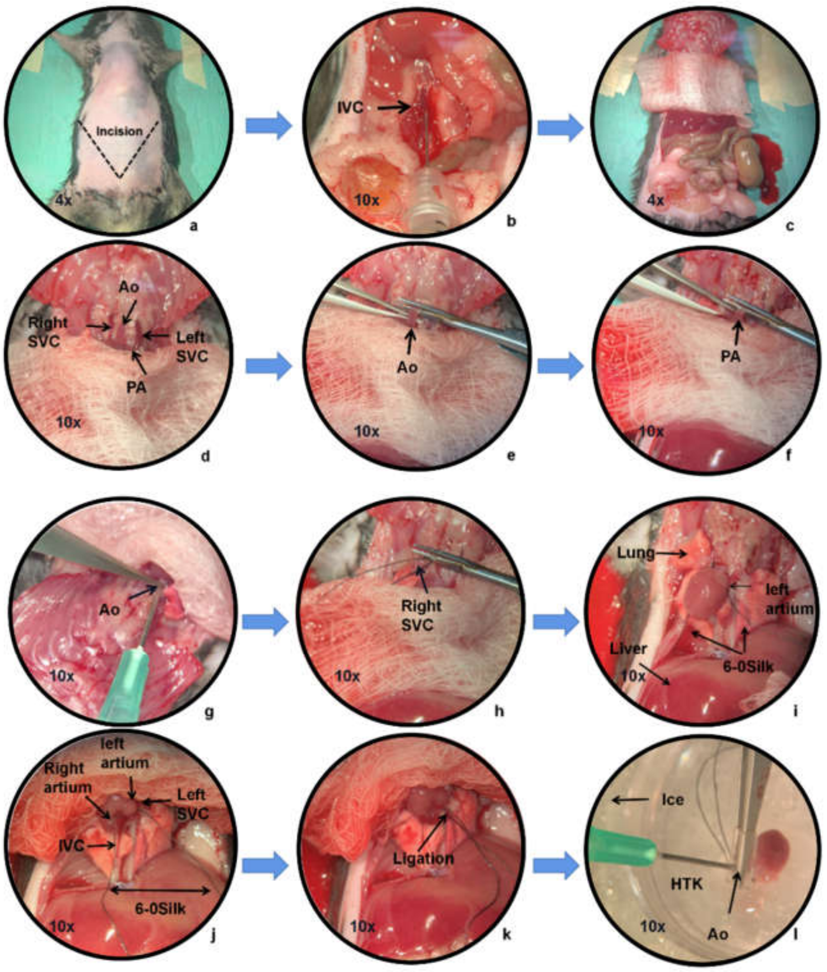

Supplementary Figure S1: Photographic illustration of the donor operation. (a) The "V" shape abdominal incision. (b) The process of heparin injection. (c) the process of the opening thoracic cavity and placement of the cold saline pad. (d) Close-up of the Aorta (AO), pulmonary artery (PA), right superior vena cava (SVC), left superior vena cava (Azygos vein). (e-f) the AO and PA cutting off. (g) HTK perfusion via ascending aorta. (h) ligation of the right SVC. (i-k) close-up of anatomical position and ligation suture placing the second ligation. (l) the second time of HTK perfusion for the isolated heart. AO: aorta; PA: pulmonary artery; IVC: inferior vena cava; SVC: superior vena cava

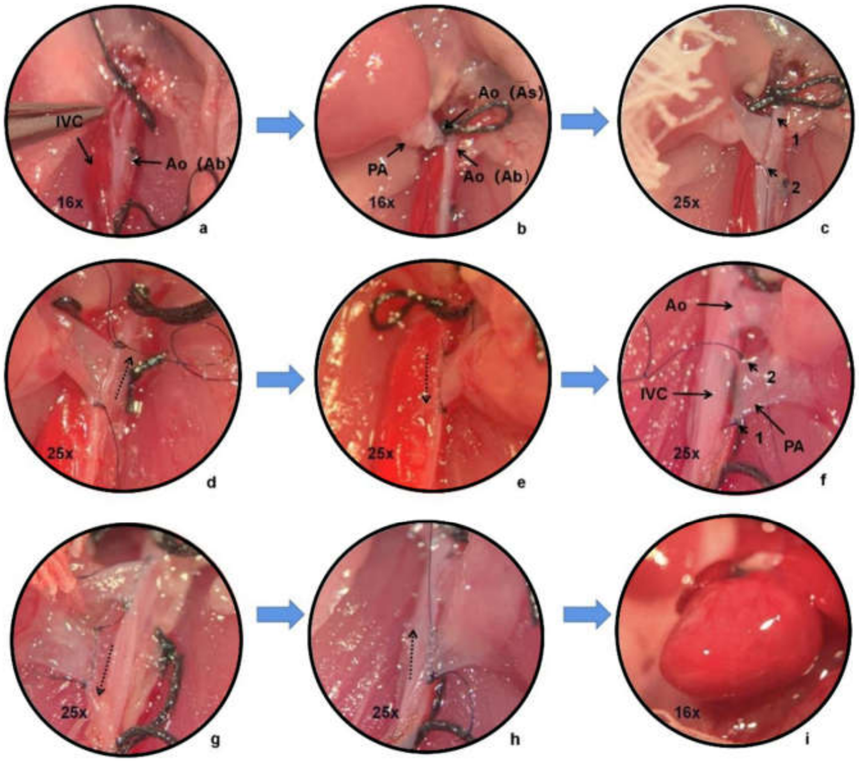

Supplementary Figure S2 Photographic illustration of the operation procedure in a recipient mouse. (a) The anatomical position of the abdominal inferior vena cava (IVC) and abdominal aorta (AO, Ab). 6-0 sutures were used to block the blood flow of the surgical area. (b) The first anchor stitch of the aorta, which is the first step for anastomosis of graft ascending aorta and recipient abdominal aorta. (c) The two anchor stitches of aorta anastomosis. Numbers are marked as anchor stitch order. (d-e) the running horizontal mattress suture of aorta anastomosis. (f) Illustration of the pulmonary artery's anchor stitches. 1 and 2 are the sequence of anchor stitches. (g-h) the running suture of the pulmonary anastomosis. Arrows represent the direction of the running sutures. (i) the graft statues after releasing blood flow.AO (As): ascending aorta; AO (Ab): abdominal aorta; PA: pulmonary artery; IVC: inferior vena cava

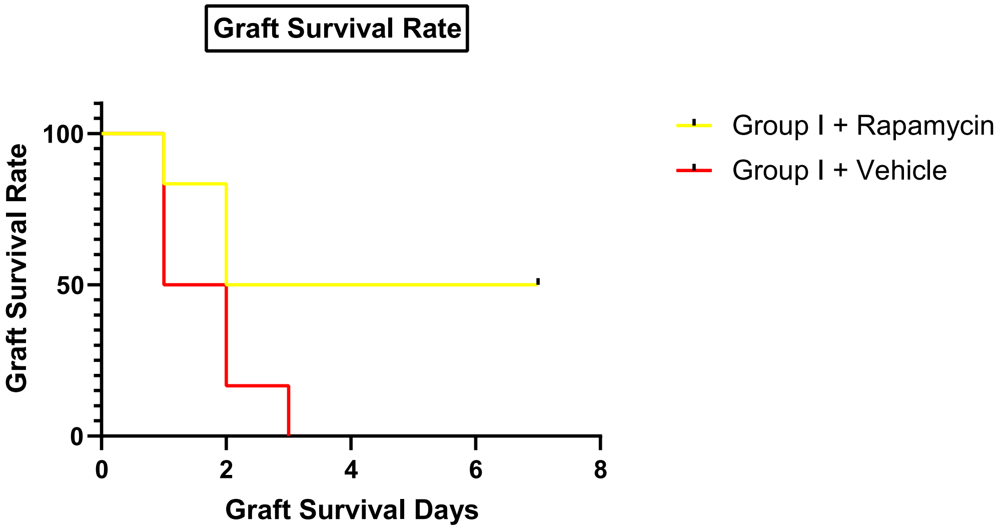

Supplementary Figure S3：Graft survival rate after 12-hours isolated heart preservation. Yellow curve represents survival rate of rapamycin (8mg/kg) treated donor heart, Red curve represents survival rate of vehicle treated donor hearts. (n=6)
